# Multiple Mechanisms Regulate Eukaryotic Cytochrome C Oxidase

**DOI:** 10.3390/cells10030514

**Published:** 2021-02-28

**Authors:** Rabia Ramzan, Bernhard Kadenbach, Sebastian Vogt

**Affiliations:** 1 Cardiovascular Research Laboratory, Biochemical-Pharmacological Center, Philipps-University Marburg, Karl-von-Frisch-Strasse 1, D-35043 Marburg, Germany; ramzan@med.uni-marburg.de; 2 Fachbereich Chemie, Philipps-University, D-35032 Marburg, Germany; 3 Department of Heart Surgery, Campus Marburg, University Hospital of Giessen and Marburg, D-35043 Marburg, Germany; vogts@med.uni-marburg.de

**Keywords:** mitochondria, energy metabolism, respiration, regulation, cytochrome c oxidase, adenine nucleotides, electron transport, efficiency, ROS generation

## Abstract

Cytochrome c oxidase (COX), the rate-limiting enzyme of mitochondrial respiration, is regulated by various mechanisms. Its regulation by ATP (adenosine triphosphate) appears of particular importance, since it evolved early during evolution and is still found in cyanobacteria, but not in other bacteria. Therefore the “allosteric ATP inhibition of COX” is described here in more detail. Most regulatory properties of COX are related to “supernumerary” subunits, which are largely absent in bacterial COX. The “allosteric ATP inhibition of COX” was also recently described in intact isolated rat heart mitochondria.

## 1. Introduction

There exists a basic difference in the regulation of ATP synthesis by oxidative phosphorylation (OxPhos) between bacteria (except cyanobacteria) and eukaryotic organisms. Aerobic bacteria grow and divide continuously under constant conditions. Higher organisms change the rates of ATP synthesis and consumption up to a factor of 10 depending on various inner and outer signals. This regulation is based on acquisition of additional regulatory protein subunits (supernumerary subunits) during evolution for the enzyme complexes of OxPhos, which in eukaryotes are located in special organelles, the mitochondria.

OxPhos includes the respiratory chain containing the enzyme complexes I (NADH: ubiquinone oxidoreductase), II (succinate dehydrogenase), III (cytochrome bc_1_ complex), IV (cytochrome c oxidase, COX), and V (ATP synthase). According to the Mitchell theory [1,2], a proton motive force Δp is involved in OxPhos as an energy-rich intermediate for the exergonic oxidation of the reducing equivalents NADH and flavin adenine dinucleotide (FADH_2_), consisting of a membrane potential ΔΨ_m_ and a pH gradient across the inner mitochondrial membrane (Δp = ΔΨ_m_ − 59·ΔpH (mV)). Δp consists mostly of ΔΨ_m_ and is generated in the respiratory chain at enzyme complexes I, III, and IV via the translocation of protons from the matrix into the intermembrane space. Degradation of ΔΨ_m_ occurs predominantly via ATP synthase and “uncouplers” of OxPhos or uncoupling proteins [3].

## 2. Cytochrome C Oxidase (COX)

Eukaryotes synthesize ATP mostly in mitochondria, with COX as the final and oxygen-accepting complex of the respiratory chain. COX represents the rate-limiting step of respiration in living cells [4,5]. In contrast, from application of the metabolic control analysis to isolated mitochondria [6,7,8], a 5–7-fold excess of COX capacity was measured over the amount required to support the endogenous respiration of isolated mitochondria [9,10,11]. Therefore, the rate-limiting and regulatory role of COX for respiration in living organisms was ignored for a long time.

With increasing complexity during evolution [12], the number of protein subunits in the COX complex increased from 2–4 in bacteria to >7 in the slime mold *Dictyostelium discoideum*, 11 in yeast, and 13 in mammals [13]. In eukaryotes the “catalytic” subunits I–III are encoded on mitochondrial DNA and synthesized in mitochondria. The additional “supernumerary” subunits are encoded on nuclear DNA and synthesized on cytoplasmic ribosomes [14]. For the transport of these subunits into mitochondria [15] and for the assembly into the 13-subunit COX complex of vertebrates, a complicated machinery is required [16].

## 3. Regulation of COX Activity by “Allosteric ATP-Inhibition”

By studying the kinetics of ferrocytochrome c oxidation in isolated COX or in mitochondria after solubilization in 1% Tween-20, full inhibition of oxygen uptake was found at high ATP/ADP ratios and low ferrocytochrome c concentrations [17]. The polarographically measured oxygen uptake at increasing ferrocytochrome c concentrations revealed a sigmoidal curve with a Hill coefficient of 2. At low ATP/ADP ratios, a normal hyperbolic curve was found. Half-maximal inhibition of activity was measured at ATP/ADP = 28 [18]. This “second mechanism of respiratory control” is independent of ΔΨ_m_ and presents a feedback inhibition of mitochondrial respiration by its final product ATP [19]. The sigmoidal curve (Hill coefficient = 2) indicates cooperativity of two substrate (ferrocytochrome c)-binding sites. These occur in the dimeric enzyme, as visualized in the first crystal structure of COX [20], since monomeric COX contains only one binding site for cytochrome c [21]. After pretreatment of solubilized mitochondria with a monoclonal antibody against subunit IV, the inhibition of COX activity at high ATP/ADP ratios was completely abolished, and further experiments indicated that ATP interacts with the matrix domain of the transmembraneous subunit IV [17]. The “allosteric ATP inhibition of COX” is absent in bacterial COX [22], which does not have the “supernumerary” subunit IV (except cyanobacteria [23]).

Regulation of COX activity by adenine nucleotides evolved early during evolution, since it occurs already in COX of cyanobacteria containing an aa_3_-type enzyme [24]. Purified COX from *Synechocystis* contains four protein subunits corresponding to subunits I, II, III, and IV of vertebrate COX. The deduced amino-acid sequence of “subunit IV” from *Synechocystis* sp. PCC6803 showed approximately 50% and 20% sequence identity to COX subunit IV from *Saccharomyces cerevisiae* and beef heart, respectively. After reconstitution in liposomes, intraliposomal ADP stimulated and ATP inhibited the oxidation of ferrocytochrome c by cyanobacteria [23].

The allosteric ATP inhibition of COX was recently also shown with intact isolated rat heart mitochondria [25]. In a previous study, it was suggested that cAMP-dependent phosphorylation of COX subunit I at Ser-441 switches on the allosteric ATP inhibition [26]. This site is located at the intermembrane space and is accessible to cytosolic calcium via the pore in the outer mitochondrial membrane. Ser-441 of COX subunit I from beef is the only amino acid of consensus sequences for cAMP-dependent phosphorylation, located at the intermembrane space [26]. Stress increases cytosolic calcium (>1 µM Ca^2+^) [27,28] and dephosphorylates this site via a Ca^2+^-activated protein phosphatase. It was suggested that the allosteric ATP inhibition of COX keeps the mitochondrial membrane potential (ΔΨ_m_) at low values (120–140 mV) and prevents formation of high amounts of ROS (reactive oxygen species) [28,29,30]. ROS have been shown to be involved in the generation of numerous diseases [31,32,33,34].

## 4. Role of Calcium in Generation of Increased ROS and ΔΨ_m_

The relationship between stress factors and the generation of ROS was unknown until recently [31,32]. We presented a regulatory mechanism explaining the formation of ROS in mitochondria [28], which includes the increase of cytosolic calcium by stress factors and the abolishment of the “allosteric ATP inhibition of COX” (see below).

Stress factors are known to increase the cytosolic calcium concentration [28,32,33], which, under resting conditions, is very low (<1 µM). Calcium represents the most important signal for mitochondrial activation [34], and this occurs via dephosphorylation of most mitochondrial proteins through calcium-dependent mitochondrial phosphatases [34]. The stress factors, which induce hyperpolarization of ΔΨ_m_, include high glucose [35,36], stress hormones [37,38,39,40,41], and psychosocial stress [42,43,44,45,46]. Furthermore, high amounts of glutamate induce mitochondrial hyperpolarization, ROS generation, and enhanced oxygen consumption in murine neuronal HT22 cells [47].

**Figure 1:**
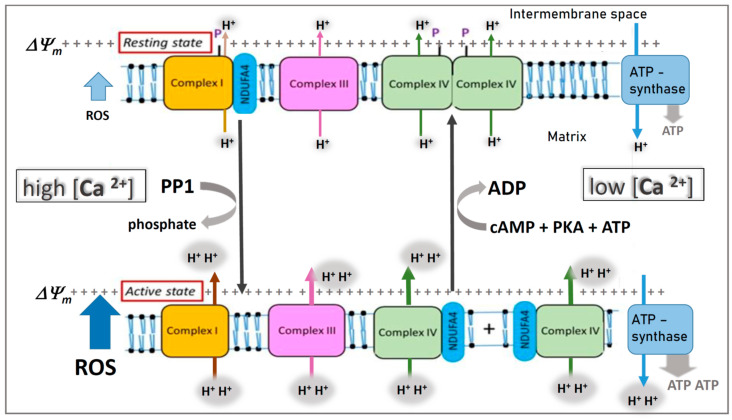
Hypothesis: Variable binding of NDUFA4 to complex I or cytochrome c oxidase (COX) in the mitochondrial respiratory chain. It is assumed that, in the resting state, phosphorylation (P) of complex I and COX (complex IV) by a cAMP-dependent protein kinase A (PKA) at low cytosolic calcium (<1 µM) stabilizes binding of NDUFA4 to complex I and induces “allosteric ATP inhibition” of dimeric COX. In the active state, stress-induced increase of cytosolic calcium (> 1 µM) dephosphorylates complex I and COX via a calcium-activated protein phosphatase (PP1), accompanied by monomerization of COX, changed binding of NDUFA4 from complex I to monomeric COX, and switching off its allosteric ATP inhibition. Note, in the “active state”, there is higher proton pumping (H^+^) and ATP synthesis, but ROS production is also increased (blue arrow). Modified from Figure 1 in [92].

The **“resting state”** of mitochondrial respiration (see Figure 1) with high efficiency requires cardiolipin to stabilize the presumed weak interaction between the two monomers in dimeric COX [17]. The crystallized structure [20] contains, instead of ADP, 10 cholate molecules per monomer, which stabilize the non-physiological dimer [48]. The weak interaction between monomers in dimeric COX under physiological conditions is supported by observations of Kyoko Shinzawa-Itoh: “*I have tried purifying cholate-free COX already. However, unfortunately, I have never succeeded in preparing COX samples enough for crystallization without using cholate*” (personal communication to B.K., 2020). The high efficiency of dimeric COX is apparently due to allosteric ATP inhibition, which keeps ΔΨ_m_ at low values [28], thus preventing proton leak of the membrane at high ΔΨ_m_ [49], which increases with increasing ΔΨ [50] and causes slippage of proton pumping in COX [51]. In addition, it was suggested that the higher efficiency of the “resting state” is based on increased proton pumping in COX with a H^+^/e^−^ stoichiometry of 2, instead of 1, which is thermodynamically possible [20]. Babcock and Wikström claimed that the H^+^/e^−^ stoichiometry of COX is constant and always 1 [52] (however, see [53]). The additional proton pumping was proposed to be related to the H-channel, identified by the Yoshikawa group in the bovine heart enzyme [54,55,56], which is absent in bacteria [57]. An H^+^/e^−^ stoichiometry of 2 in COX was in fact measured by the Lehninger group in isolated rat liver mitochondria [58,59,60,61].

The **“active state”** of mitochondrial respiration (without allosteric ATP inhibition) (see Figure 1) is characterized by maximal rates of COX activity and ATP synthesis. This state has partly elevated ΔΨ_m_ [30], accompanied by low efficiency. It is switched on under stress, including psychosocial stress [28], via increased cytosolic calcium concentrations, which dephosphorylate the dimeric COX resulting in monomeric COX [25], which is bound to NDUFA4 for stabilization [62]. These results exclude NDFUA4 as a permanent 14th subunit of COX. The activity of monomeric COX is supported by results obtained with purified COX. Removal of subunit VIb, which participates in the dimerization [26], increases COX activity [63]. In addition, without the allosteric ATP inhibition of COX, ΔΨ_m_ could rise above 130 mV, accompanied by proton leak [49] and slippage in COX [51], decreasing the efficiency of OxPhos. Although the respirasome (supramolecular structure I_1_III_2_IV_1_) contains only monomeric COX, the ordered cluster structure of the complete OxPhos system could allow a fast monomeric/dimeric configuration change. Thus, the establishment of “allosteric ATP control” mirrors the molecular composition [64].

In isolated mitochondria, supplemented with succinate, pyruvate, or glutamate, ADP stimulates oxygen consumption (state 3), whereas, after its conversion into ATP, respiration decreases (state 4) and ΔΨ_m_ increases to 180–200 mV. Moreover, isolated and reconstituted COX in liposomes could create a ΔΨ up to 225 mV [51]. The ratio of the rate of respiration at state 3 to state 4 was named “respiratory control”. In isolated mitochondria at state 4, 2% of total consumed oxygen was found to be converted into H_2_O_2_ [65]. This high amount of ROS (reactive oxygen species, mainly superoxide radical anion O_2_^.−^ and H_2_O_2_) is deleterious for life and has been shown to be involved in the generation of numerous diseases [66,67,68,69,70]. In contrast, small amounts of ROS, produced by various oxidases [71,72], have signaling functions [31,73].

This high amount of ROS is produced in mitochondria mainly at complexes I–III [74,75], but not in COX, due to the unique structure of the oxygen-binding site in subunit I, composed of heme a_3_, Cu_B_, and a tyrosyl-group, allowing simultaneous transfer of four electrons to O_2_, thus preventing the formation of intermediate ROS compounds [26]. It was found that mitochondrial ROS generation increases exponentially with increasing ΔΨ_m_ values above 140 mV [76,77,78]. Furthermore, high NADH/NAD^+^ ratios lead to the formation of O_2_^−^ [73], which is rapidly converted into H_2_O_2_ by superoxide dismutases in the matrix and in the intermembrane space [79]. High and deleterious ΔΨ_m_ values above 140 mV are not necessary for the synthesis of ATP, because the ATP synthase is already saturated and maximal at 100–120 mV [80].

Fortunately, in living cells, mitochondrial ΔΨ_m_ values are normally below 140 mV (see [81]). However, under various stress conditions, an increase in ΔΨ_m_ to high and deleterious values was described. This mostly transient increase of ΔΨ_m_ was named mitochondrial “hyperpolarization” and is often followed by cell apoptosis [82,83].

Inhibition of ΔΨ_m_ by ATP was also measured directly in isolated rat liver mitochondria using a tetraphenyl phosphonium electrode [84]. Above 140 mV, the ROS production in mitochondria increases continuously [26,76,77]. While low ROS concentrations have regulatory functions in cells [31,73,75], high amounts, produced in mitochondria under stress [28], are known to cause multiple diseases [65,66,67,68,69] and to accelerate aging [72,85,86].

Allosteric ATP inhibition requires dimeric COX, based on its cooperativity of substrate action [25]. Monomeric COX, as isolated in nonionic detergents, is rather stable, while the dimer form is intrinsically unstable and dissociates into monomers at increased detergent concentration [87]. In isolated bovine heart mitochondria, more than 85% was found to be monomeric [88]. Bile acids stabilize the dimeric form [89] by exchanging bound ADP with cholate [90].

The feedback inhibition of COX by ATP, discovered 23 years ago, was not recognized by the scientific community, because it was generally not found in isolated mitochondria for two reasons: (i) measurements in mitochondria were usually not performed in the presence of ATP and high ATP/ADP ratios; (ii) the mechanism is switched off under stress conditions via Ca^2+^-activated dephosphorylation of COX. The phosphorylation site is located at the cytosolic side of COX subunit I [91], and it is essential for allosteric ATP inhibition.

After dephosphorylation of this site by a Ca^2+^-activated protein phosphatase, allosteric ATP inhibition is switched off. Re-phosphorylation by a cAMP-dependent protein kinase switches it on again [25]. These observations were made with the isolated enzyme, partly reconstituted in liposomes [28,90,93]. The reversible switching on and off of allosteric ATP inhibition was also shown recently with intact rat heart mitochondria [25]. In this study, a very low concentration of calcium (1–10 µM) was sufficient to switch off allosteric ATP inhibition. Various stress signals increase the cytosolic Ca^2+^ concentration and activate a Ca^2+^-dependent protein phosphatase, located at the intermembrane space, leading to dephosphorylation of COX with subsequent loss of allosteric ATP inhibition, increase in ΔΨ_m_, and formation of ROS [28] (see Figure 1).

In conclusion, we suggest that allosteric ATP inhibition of COX is continuously changing in all mitochondria in order to optimize the amount and efficiency of energy (ATP) generation in eukaryotic cells according to the actual requirements (see Figure 1).

## 5. Regulation of COX via Reversible Phosphorylation

A further regulation of COX activity occurs via reversible phosphorylation of protein subunits [91,92,94,95]. Using mass spectrometry, 18 different phosphorylation sites were identified in COX subunits [91]. The specific functions of phosphorylation sites, however, were identified only in a few cases (see above). A review of phosphorylation sites was presented by Covian and Balaban [95] and Hüttemann et al. [96]. Selective protein kinase A (PKA)-dependent phosphorylation of subunits I, IV-1, and Vb was found under hypoxic stress in rabbit heart [97]. Interestingly, in cytochrome c, the reducing substrate of COX is also reversibly phosphorylated (at five positions), which influences the catalytic property of COX [96].

## 6. Regulation of COX via Expressing Supernumerary Subunit Isoforms

Regulation of COX activity via expressing isoforms of supernumerary subunits in the complex of 13 total subunits was first described in 1982 for subunits VIa, VIIa, and VIII [98]. Isoforms of supernumerary subunits occur in tissue-, developmental-, and species-specific forms. The identification of isoforms for subunits VIa, VIIa, and VIII occurred in skeletal muscle (H-isoform) and in non-skeletal muscle tissues (L-isoform), including smooth muscle [99], as shown by different runs in SDS-PAGE and by different N-terminal amino acid sequences [98]. The two genes for an isoform were published first in 1988 for subunits VIa (VIa-H = heart isoform, VIa-L = liver-isoform [100]). In most tissues, the liver isoform was found to occur (VIa-L, VIIa-L, VIIIa-L), which differs from the isoforms occurring in muscle tissue (VIa-H, VIIa-H, VIII-H), except in smooth muscle [99]), indicating different genes of these isoforms in skeletal muscle tissue.

Meanwhile, six different isoforms were identified for supernumerary COX subunits [101]. These include subunit IV (IV-1 + IV-2 [102] + IV-3 [103]). Allosteric ATP inhibition of COX is active in most cell types which express subunit IV-1. Isoform subunit IV-2 was found to be expressed in human cell lines under hypoxia [102,104]. Moreover, in isolated astrocytes and cerebellar granule cells, subunit IV-2 is expressed under hypoxic conditions accompanied by an abolition of the allosteric inhibition of COX by ATP [105]. The third isoform for COX subunit IV (Coxfa4l3) was found to occur during spermatogenesis [103]. Further isoforms occur for subunit VIa (VIa-H + VIa-L), VIb (VIb-1 and VIb-2, which appears to be testis-specific [106]), VIIa (VIIa-H + VIIa-L + COX-7AR [107]), VIIb (VIIb-1 + VIIb-2 [108]), and VIII (VIII-H + VIII-L + VIII-3 [109]).

Interestingly, in the other complexes of OxPhos (complexes I, II, III, and V), no isoforms of supernumerary subunits were found.

A specific expression of supernumerary COX isoforms concerns developmental-specific isoforms. During fetal development of mammalian embryos, the liver-type COX subunits VIa-L and VIIa-L are expressed, before switching to expression of the respective heart-type subunits during birth [110,111].

## 7. Regulation of COX via Binding Small Metabolites, Proteins, and Ligands and Deacetylation of Subunits

Various metabolites have been described to bind to COX and change its activity. **Copper** (**Cu**) is an essential trace element required for the normal development of living organisms. Copper-dependent enzymes such as ceruloplasmin, superoxide dismutase SOD1 and SOD3, the group of metallothionein proteins, and COX are present at all stages of gametogenesis, as well as in the somatic cells of the testis. Due to its redox potential, copper is a cofactor in many enzymes responsible for important processes in cells. Copper is a very reactive element and, in its free state, it can trigger the production of large amounts of free radicals, which consequently lead to the damage of proteins and DNA [112]. Hepatic expression of the Cu chaperones antioxidant 1 copper chaperone and cytochrome c oxidase copper chaperone (COX17) was decreased during iron deficiency, while the expression of the genes of zinc metabolism was unaltered [113]. the anion-binding behavior of the **magnesium/manganese** (Mg/Mn) site in cytochrome c oxidase has a possible role in proton pumping. Due to its close proximity and a shared ligand, oxidized Cu(A) is spin-coupled to the Mn(II) ion. This new observation of anion binding at the Mg/Mn site is of interest in terms of accessibility of the buried site and its potential role in redox-dependent proton pumping [114]. Moreover, the buried Mg/Mn site in COX is important for water ligands. Possibly, it represents a pathway for the exit of protons or water produced during turnover [115]. Inhibition of COX is viewed as a primary mode of cytotoxic **hydrogen sulfide** (**H_2_S**). However, studies conducted over the last two decades unveiled multiple biological regulatory roles of H_2_S as an endogenously produced mammalian gaseous transmitter. H_2_S serves as a stimulator of electron transport in mammalian mitochondria by acting as an electron donor, with sulfide/quinone oxidoreductase (SQR) being the immediate electron acceptor, and it stimulates mitochondrial ATP production [116]. A conserved **bile acid**-binding site (BABS) was crystallographically defined in the membrane domain of mammalian COX and COX from *Rhodobacter sphaeroides*. Diverse amphipathic ligands were shown previously to bind to this site and affect the electron transfer equilibrium between heme a and a_3_ cofactors by blocking the K proton uptake path. Identified candidate ligands include steroids, nicotinamides, flavins, nucleotides, retinoic acid, and thyroid hormones, which are predicted to make key protein contacts with the residues involved in bile acid binding [117]. **Deoxycholate** binds with its carboxyl group at the entrance of the K path; thus, this conserved steroid binding site could reveal a regulatory site for steroids or structurally related molecules that act on the essential K proton path and enzyme activity [118]. Cholate can affect the splitting up of COX into two conformational states. Following the kinetics of cyanide binding to the oxidized enzyme, different fractions of “slow” and “fast” conformations are found. The structural relationships between the known cholate-binding site and the binuclear cytochrome a_3_-CuB site, as well as the variation in the occupancy of this binding site with cholate or nucleotides, may modify the reactivity of the oxidized binuclear center toward **cyanide** [119]. 3,5-Diidothyronine (3,5-T2) binds to COX subunit Va and abolishes allosteric ATP inhibition of COX [120]. Another metabolite is palmitate, which decreases the H^+^/e^−^ stoichiometry of proton pumping of non-muscle COX [121]. The binding of proteins to COX, including voltage-dependent anion channel (VDAC), epidermal growth factor receptor (EGFR), HIG1 domain family member 1A (Higd1a), B-cell lymphoma 2 protein (BCL-2), a hepatitis B viral protein (HBx), mitochondrial nuclear retrograde regulator 1 (MNRR1), Coiled-coil-helix-coiled-coil-helix domain containing 2 protein (CHCHD2), amyloid-β protein, Nitric oxide synthase 1 (NOS-1), NDUFA4, and NADH dehydrogenase (ubiquinone) 1 alpha subcomplex, 4-like 2 (NDUFA4L2), was discussed in former publications [101,107].

Mitochondrial complexes are prone to sirtuin (Sirt)3-mediated deacetylation modification. COX subunit I was discovered as a new deacetylation target of Sirt3, indicating that the Sirt3/MT-CO1 axis is a promising therapy target of stress-related diseases [122].

## 8. Overexpression of COX Subunits during Ischemic Injury, Cancerogenesis and Regulation via Forming Supercomplexes

Upregulating the expression levels of mitochondrially encoded NADH dehydrogenase 1 (MT-ND1) and COX subunit I (MT-CO1) in tachypacing cardiomyocytes resulted in increased ATP content and superior cell viability, whereas the expression levels of NADH ubiquinone oxidoreductase core subunit 1 (NDUFS1) and COX subunit VIc (COXVIc) had no effect [123]. Cytochrome c oxidase subunit Va (COXVa) is involved in maintaining normal mitochondrial function. Newly established transgenic mice with systemic COXVa overexpression resulted in the improvement of spatial recognition, memory and hippocampal synaptic plasticity, recovery of hippocampal CA1 dendrites, and activation of the BDNF/ERK1/2 (brain-derived neurotrophic factor/extracellular signal-regulated kinase) signaling pathway in vivo. COXVa in the hippocampus plays a vital role in aging-related cognitive deterioration via BDNF/ERK1/2 regulation, suggesting that COXVa may be a potential target for antisenescence drugs [124]. COXVa overexpression protects cortical neurons from hypoxic ischemic injury in neonatal rats associated with triosephosphate isomerase upregulation [125]. COXVb subunit is linked directly to stress regulatory networks. Copper/zinc superoxide dismutases possess the ability to enhance copper sulfate stress response and induction of messenger RNA (mRNA) transcription levels and microRNA [126]. In human hepatocellular carcinoma (HCC) tissues, 11 out of the 13 mitochondrial DNA (mtDNA)-encoded genes exhibited decreased mRNA levels and five genes displayed decreased protein levels, including the cytochrome B and MT-CO2 genes. After overexpression of mitomiR-181a-5p, cytochrome B and MT-CO2 levels were reduced in HCC cells, and the ΔΨ_m_ maintained by the electron transport chain (ETC) was decreased [127]. A systematic study of the overexpression of proteins and genes in tumor cells resulted in various publications. Somatic mutations within mitochondrial DNA-encoded MT-CO1 are frequent in various cancer types. Reactive oxygen species generated in cells overexpressing MT-CO1 variants acted as key effectors mediating differential expression of apoptosis and DNA damage pathway-related genes [128]. Overexpression of COXVIb1 protected against ischemia/reperfusion-induced neuronal injury in rat hippocampal neurons and relieved hypoxia/reoxygenation injury of neonatal rat cardiomyocytes [129,130]. Overexpression of COXVIIa1 suppressed cell proliferation and colony formation ability, as well as promoted cell apoptosis, in human non-small-cell lung cancer cells. COXVIIa1 holds a key position in regulating the development and progression of lung cancer by affecting autophagy [131]. In some reports, an increased correlation between the overexpression of COX subunit Va and/or Vb and the growth rate of various tumors was found [132,133,134,135,136,137,138]. The molecular basis for this relationship, however, remained unsolved.

Similar to the association of two COX monomers to the dimer, the complexes of the respiratory chain (CI–CIV) can associate to multiple complexes called “supercomplexes”. A well-known supercomplex is the “respirasome” (I_1_III_2_IV_1_). Their properties were described in review articles [139,140,141,142]. The links among mitochondrial dynamics, cristae remodeling, and supercomplex formation are presented therein: how mitochondrial structure can regulate bioenergetics, as well as the impact of mitochondrial dynamics and cristae shape on oxidative metabolism, the respiratory efficiency, and redox state. For example, CI (complex I) forms a supercomplex with CIII_2_ and CIV (SC I_1_+III_2_+IV_1_), known as the respirasome), as well as with CIII_2_ alone (SC I + III_2_). CIII_2_ forms a supercomplex with CIV (SC III_2_ + IV_1_), and CV alone forms dimers (CV_2_).

## Data Availability

Data sharing not applicable No new data were created or analyzed in this study. Data sharing is not applicable to this article.
